# Using Shadow Page Cache to Improve Isolated Drivers Performance

**DOI:** 10.1155/2015/896519

**Published:** 2015-02-28

**Authors:** Hao Zheng, Xiaoshe Dong, Endong Wang, Baoke Chen, Zhengdong Zhu, Chengzhe Liu

**Affiliations:** ^1^Department of Computer Science and Technology, Xi'an Jiaotong University, Xianning West Road No. 28, Xi'an 710049, China; ^2^State Key Laboratory of High-End Server & Storage Technology, Xinluo Street No. 1799, Jinan 250000, China

## Abstract

With the advantage of the reusability property of the virtualization technology, users can reuse various types and versions of existing operating systems and drivers in a virtual machine, so as to customize their application environment. In order to prevent users' virtualization environments being impacted by driver faults in virtual machine, Chariot examines the correctness of driver's write operations by the method of combining a driver's write operation capture and a driver's private access control table. However, this method needs to keep the write permission of shadow page table as read-only, so as to capture isolated driver's write operations through page faults, which adversely affect the performance of the driver. Based on delaying setting frequently used shadow pages' write permissions to read-only, this paper proposes an algorithm using shadow page cache to improve the performance of isolated drivers and carefully study the relationship between the performance of drivers and the size of shadow page cache. Experimental results show that, through the shadow page cache, the performance of isolated drivers can be greatly improved without impacting Chariot's reliability too much.

## 1. Introduction

By transparently integrating a variety of hardware resources, the virtualization technology can provide reliable and customizable operating environment for users. In this environment, a user can directly reuse many software resources without any change, such as a variety of the operating systems (OS). Relative researches have shown that driver faults are the main factor causing OS crashes [1–4], and this reliability problem also exists in the user customized virtual environment. Though the isolation property between virtual machines (VM) can be used to improve the reliability of the entire server [5–7], it cannot isolate driver faults in a VM. In order to solve the driver's reliability problem inside the VM, Chariot [8] provides fine-grained isolation environment for drivers inside the VM using the shadow page and access control table (ACT). It establishes a driver's ACT which records the memory the driver can write and captures a driver's write operations by setting shadow pages corresponding to the entire kernel address space of the VM where the driver is inside (referred to as VM's shadow pages) to read-only in the virtual machine monitor (VMM). Then, Chariot can judge the correctness of a driver's write operations and prevent the spread of driver faults.

However, Chariot needs to timely capture driver's write operations for correctness judgments; this often needs to keep VM's shadow pages to read-only. Even an allowed write operation, the permission of the written shadow page should be reset to read-only after it finished. If there are frequent interactions between a driver and the kernel, simply setting the VM's shadow pages to read-only may cause great performance loss. To this end, this paper mainly studies how to effectively capture the driver's write operations to avoid large performance loss under the condition of ensuring the driver fault isolation. We propose the algorithm of shadow page cache for the driver's write operation capture (SCDWC). According to the principle of locality, it caches the information of shadow pages that are recently written and delays setting write protections of these shadow pages to avoid their permissions being repeatedly opened and closed.

To this end, this paper mainly studies how to effectively capture driver's write operations without impacting a driver's performance too much, while ensuring a high reliability property of Chariot. We study the single-level shadow page caching for a driver's write operation capture (SCDWC) algorithm: according to the principle of locality, this algorithm caches the information of recently written shadow pages and delays setting the write protection of these shadow pages to avoid page faults caused by a lack of write permissions.

Considering the interface of the network driver is the most complex and its interactions with the kernel are the most frequent than other type of drivers, the write operation capture of the network driver also has the largest impact on its performance. This paper uses the* e1000* network driver to study the performance and reliability of the algorithm, so as to determine the optimal parameter of the proposed algorithm in* KVM* environment. The experimental results show that the new write operation capture algorithm significantly improves the performance of Chariot, ensures just a slight descent of the isolation rate, and further improves the practicability of Chariot.

The layout of this paper is as follows.  Section 2 of the paper will introduce the architecture of Chariot.  Section 3 describes the traditional approach in Chariot to capture write operations.  Section 4 describes the approach of SCDWC algorithm and verifies its efficiency.  Section 5 is the experimental evaluation of SCDWC for different drivers.  Section 6 is the optimization methods used in related reliability solutions. The last section is the conclusion.

## 2. Chariot Architecture

**Figure 1:**
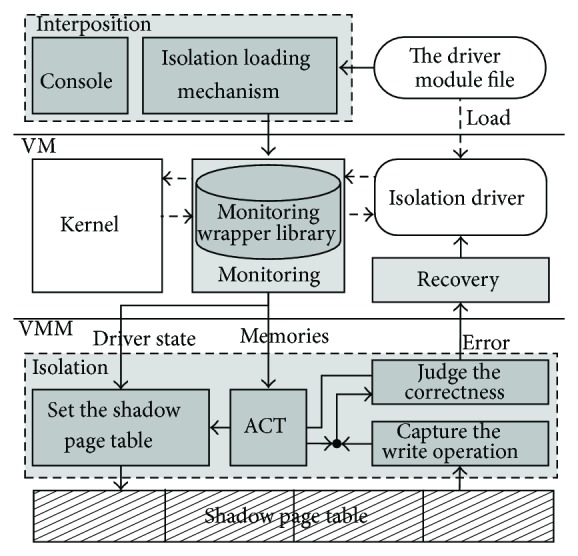
The architecture of Chariot.

Chariot is an architecture that isolate drivers inside the VM, which has the following goals: (1) efficient isolation which can effectively isolate drivers' write operation faults; (2) complete transparency, without modifying the VM kernel and drivers; (3) good scalability and portability, which can easily isolate a new driver and port itself to different versions of VM kernels; (4) little performance loss, not impacting the performance of drivers' normal operations too much. The detail components of Chariot architecture are shown in Figure 1, including four components running on different privilege levels: the interposition component, the monitoring component, the isolation component, and the recovery component.

The interposition component provides operation interfaces to users; the most important part is the isolation loading mechanism for drivers. This mechanism can connect an isolated driver to monitoring wrappers which are in the monitoring component, in the case of ensuring transparency. Then the loaded driver will be monitored by Chariot.

The monitoring component is used for transparently tracing isolated drivers. It mainly has three tasks: (1) obtain memory information which is needed by a driver's normal operation; (2) obtain all kinds of running states of a driver; (3) get the trusted kernel's code range relative to a driver.

The use of the isolation component is to provide a special operation environment, which just has the smallest set of memory resources that a driver needs and is composed of an isolated driver's ACT and the write protection of VM's shadow pages. The correctness of each write operation should also be scrutinized by this component.

The recovery component is mainly used to handle a variety of driver faults and rapidly recover the faulty driver in the case of ensuring the normal operation of VM kernels. According to the type of driver faults and user requirements, it can take different levels of recovery strategies, such as unload a faulty driver, or reload a new driver.

The goals of Chariot cannot be achieved unless all these components cooperate with each other correctly. The connection between a driver and monitoring wrappers in the monitoring component is established by the interposition component. Thereafter, monitoring wrappers are inserted between an isolated driver and the kernel transparently, and the driver is running in the isolated mode with memory usage being monitored timely. Also its memory information is reported to the isolation component to update a driver's ACT. The isolation component captures the driver's write operations by setting VM's shadow pages to read-only and examines their correctness with the help of the driver's ACT. If a driver failure is detected, the recovery component is triggered to recover the faulty driver.

## 3. The Approach of Write Operation Capture

**Figure 2:**
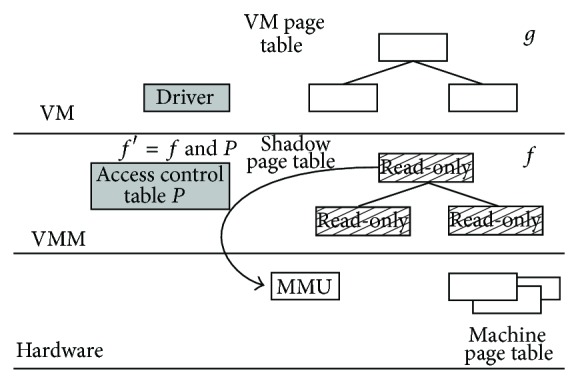
The implement of two-level page table translation in full virtualization technology.

In full virtualization technology, the translation from the virtual address of a VM instance (Guest OS) to the physical address of its Host OS is realized by a two-level page table, as shown in Figure 2. The first level is in the Guest OS; it translates the virtual address of the VM to the physical address of the VM, as the translation *g* shown in Figure 2. The second level is in the Host OS; it translates the physical address of the VM to the physical address of the Host OS, as the translation *f* shown in Figure 2. If the VM executes a write operation, it needs to combine both translations, as *f* · *g*. Then the memory management unit (MMU) will complete the remaining work.

When the driver in the VM triggers a write operation, its eventual execution will go through the second level page table (also known as the shadow page table) *f*. In other words, the page table that records the actual physical address is the translation *f* in the VMM. If this page table is set to read-only, any write operation will trigger a VMM page fault exception and then enter the page fault exception handler to selectively open the permission of read-only pages according to the exception reason. Therefore, if the shadow page table *f* of the VM where the isolated driver is inside is set to read-only, write operations of this VM can be effectively captured. Chariot uses this approach to protect a VM's shadow pages and combines it with the ACT (*P* in Figure 2) to determine the correctness of a driver's write operations, so as to detect isolated driver's write operation faults. After a legitimate write operation finished, Chariot will reclose the write permission of the opened shadow page, so that write operations can be sustainedly captured. This algorithm of capturing write operations is a traditional algorithm of the driver's write operation capture, referred to as DWC algorithm.

The detail of the DWC algorithm is described in Algorithm 1.* S* denotes the whole set of a VM's shadow pages (from 1 to *m*);* driver_state* currently indicates whether a driver is called or not;* rip* is the instruction of a write operation;* TRUST_KERNEL* denotes the trusted kernel's code range. When the driver is called, Chariot calls* WRITE_CAPTURE_PREPARE* function to set the VM's shadow pages to read-only to capture write operations (Line (3)). When a write operation of the VM triggers a page fault exception, the page fault handler will determine whether or not to open the write permission of this shadow page by* WRITE_CAPTURE_JUDGE* function (Line (7)–(15)). When a write operation is finished, the handler calls* WRITE_CAPTURE_RESET* function. If the driver is called at this time, the write permission of the opened shadow page will be closed immediately (Line (18)–(20)).

## 4. Shadow Page Cache Algorithm

### 4.1. Problem

**Figure 3:**
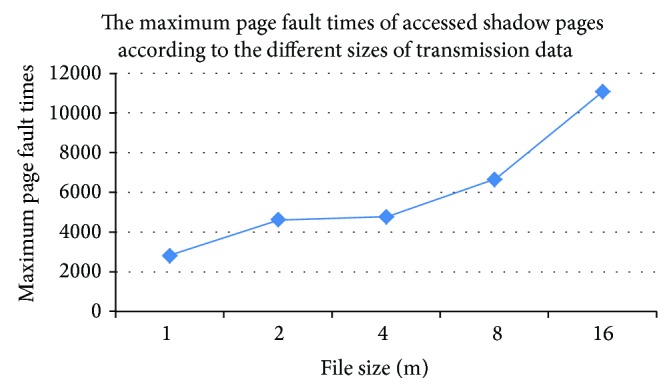
When a driver which is isolated by Chariot with DWC algorithm transmits different sizes of data, the distribution of the maximum page fault times of accessed shadow pages. The maximum page fault times mean the maximum number of page faults in the whole shadow pages.

If a driver infrequently interacts with the kernel, the performance loss caused by using DWC algorithm to capture a driver's write operations is accepted [8]. But if a driver frequently interacts with the kernel, it will bring significant performance loss. When the* e1000* network driver, which is isolated by Chariot with DWC algorithm, transmits data from 1 M to 16 M, the maximum page fault times of accessed shadow pages are shown in Figure 3. With the increasing size of the transmitted data, the maximum page fault times of a shadow page also increase. When the data size is larger than 16 M, the size of the file, which records the page fault times of all shadow pages, sharply increases. Thereby, the memory which is needed to analyze the file is too large; our ordinary PC cannot handle this to get the final analysis result. In order to better understand the distribution of page faults of shadow pages, we choose the 1 M data as an illustration. The total times of captured write operations are 96,700, and the total number of accessed shadow pages is 4751. Among them, there are 14 pages whose page fault times are larger than 1000, and these pages' page fault times are 26.91% in all pages' page fault times. In addition, there are 198 pages whose page fault times are larger than 100 and smaller than 1000, and these pages' page fault times are 52.70% in all pages' page fault times. The maximum page fault times of these pages are 2564, and the minimum times are 1. The performance loss of the network driver caused by frequent page faults is larger than 90%. The main reason is that the write permission of some shadow pages are repeatedly opened and closed. We defined this kind of phenomenon as “page fault shaking phenomenon” (PFSP). Therefore, if PFSP can be reduced, the performance of the isolated driver can be improved.

### 4.2. Approach

According to the principle of locality, the shadow page which has been accessed just now is likely to be accessed again recently. To this end, a structure of shadow page cache can be used to record shadow pages' information which has been accessed recently, and the write permission of these shadow pages will be delayed to reset to read-only. Then PFSP can be avoided. This method of using the shadow page cache to improve drivers' performance is called the algorithm of shadow page cache for write operation capture, referred to as SCDWC algorithm.

**Figure 4:**
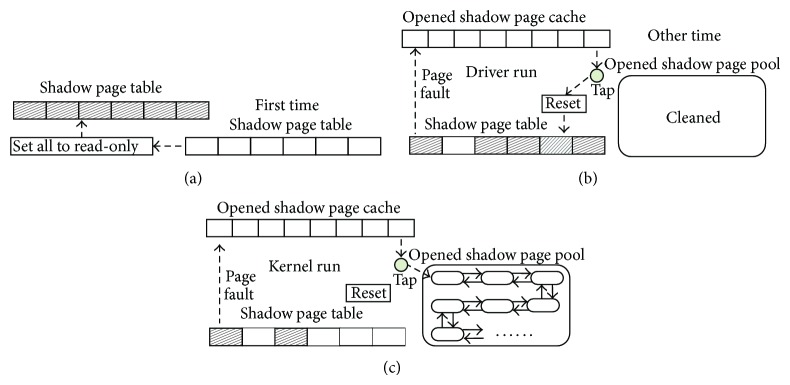
The principle diagram of SCDWC algorithm.

The length of the shadow page cache is defined as *C*. As shown in Figure 4, SCDWC algorithm is described as follows.

When an isolated driver is called at the first time, Chariot sets VM's shadow pages to read-only for drivers' write operations capture, as shown in Figure 4(a).

During the running of an isolated driver, an opened shadow page's information is pushed into the opened shadow page cache. The cached shadow page will temporarily not be reset to read-only after writing. If the cache is full, the oldest cached shadow page's information will be popped and reset this page to read-only, as shown in the left part of Figure 4(b). Thereby, the cached shadow page will only be set to read-only after *C* times of page faults.

When the driver is not called, most shadow pages' permission is still read-only. Since setting the entire shadow page table to writeable is time-consuming, which has adverse impact to the performance, we still take the page fault into account to open the write permission as needed. For a shadow page used by the kernel is also likely to be used by the subsequently called driver, in order to avoid PFSP, an opened shadow page is still pushed into the opened shadow page cache, but the popped cache is pushed to the opened shadow page pool, as shown in Figure 4(c). Because now the driver is not called, we do not need to ensure capture each write operation and just need to record opened shadow pages' information. Once the driver is called again, only shadow pages in the pool have to be set to read-only, which also reduces the permission setting overhead and avoids PFSP.

Finally, when the driver is called at the second time or more, the opened shadow pages have already been recorded in the pool and the driver's write operations can be captured by just setting these pages to read-only, as shown in the right part of Figure 4(b). The process of opening and resetting the permission of shadow pages is still the same as the first time, as shown in the left part of Figure 4(b).

The detail of SCDWC algorithm is shown in Algorithm 2. *S* and* driver_state* are the same as DWC;* SC* denotes the opened shadow page cache;* pSC* denotes a location pointer that points to the current pushed position in* SC*;* SP* denotes the opened shadow page pool;* driver_call_time* denotes the called times of an isolated driver. When the kernel calls the driver, SCDWC algorithm still sets the permission of shadow pages by* WRITE_CAPTURE_PREPARE* function, in order to capture write operations. Unlike DWC algorithm, it makes a judgement according to the driver's calling times* driver_call_time* and decides whether to set the whole shadow pages *S* to read-only or just set the shadow page pool* SP* to read-only (Line (2)–(10)). When a VM's page fault occurs, the same to DWC algorithm,* WRITE_CAPTURE_JUDGE* function is called to determine whether or not to open the write permission. Different to DWC algorithm,* WRITE_CAPTURE_RESET* function is called after the write operation, but SCDWC algorithm will push an opened shadow page to the position* pSC* of the shadow page cache* SC* and increase the position pointer* pSC* (Lines (13)–(15)). Finally, according to the driver's running state* driver_state*, the popped old shadow page (Line (17)) will be reset to read-only or sent to the shadow page pool* SP* (Lines (18), (20)).

### 4.3. Verification

**Figure 5:**
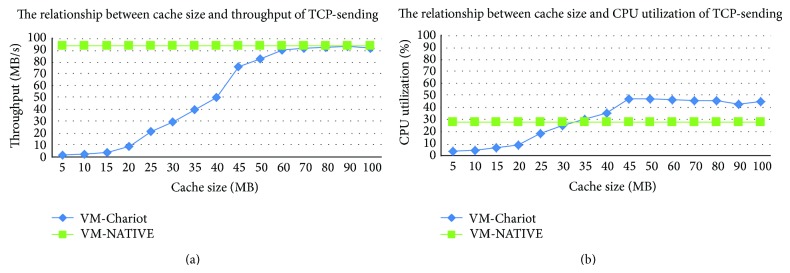
The sending performance of TCP benchmark impacted by different cache sizes.

**Figure 6:**
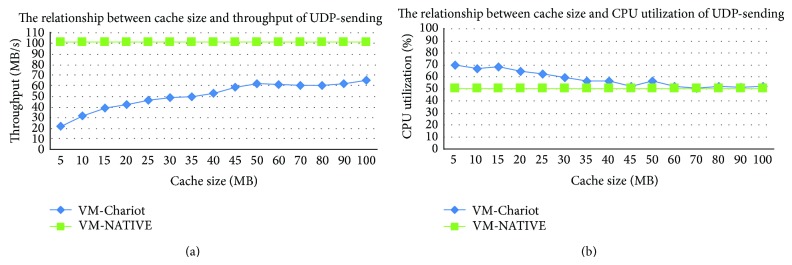
The sending performance of UDP benchmark impacted by different cache sizes.

In order to improve the performance, the size *C* of the shadow page cache cannot be too small. At the same time, in order to ensure that the driver's write operations can be effectively captured, the total number *C* of the opened shadow page caches cannot be too large. Thereby, we need to study the impact between the driver's performance and reliability by different cache sizes *C*. In the case of different cache sizes *C* from 5 to 100, the sending performance of TCP and UDP of* e1000* driver is tested using* netperf* benchmark and shown in Figures 5 and 6. For the receiving performance of* e1000* driver is not greatly impacted by different sizes *C*, we do not show it here.

As shown in Figures 5 and 6, with the increase of the shadow page cache size *C*, the throughput of the isolated driver is gradually increased, the CPU utilization is gradually decreased, and eventually both of them reach a relatively stable level. In Figures 5 and 6, when cache size *C* is 60, the sending throughput of TCP and UDP of the isolated network driver is nearly to the throughput of the native driver. Since then, continuing to increase the cache size *C*, although the throughput of the isolated driver is still slightly increased, the change is not obvious. Moreover, when *C* is 60, the increase of the isolated driver's CPU utilization is limited and is within an acceptable range.

**Figure 7:**
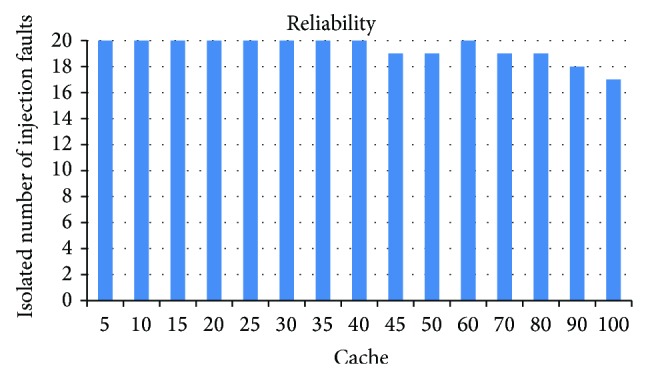
Chariot's isolation efficiency impacted by different cache sizes *C* of SCDWC algorithm.

Relative to the VM's shadow pages, 60 opened shadow pages are fairly small, which even can be ignored and almost has no impact on the efficiency of capturing write operations. In order to prove the inference, we select 20 injected driver faults which can be isolated under DWC algorithm and test whether Chariot with SCDWC algorithm can also isolate these faults. The isolation efficiency of 20 driver faults impacted by different cache sizes *C* is shown in Figure 7. It can be seen that the isolation efficiency is not obviously effected and only has a slight impact when the shadow page cache size *C* is greater than or equal to 80. Therefore, choosing 60 as the optimal parameter of the cache size *C* in SCDWC algorithm is reasonable; it just has little influence on the isolation efficiency of Chariot.

## 5. Evaluation

In this section, we apply SCDWC algorithm to other drivers and test their performance and reliability, to further verify its efficiency. We choose six different drivers to test, as follows:* e1000*,* rtl8139*,* usb_storage*,* sd_mod*,* ens1370,* and* intel8x0*.

### 5.1. Performance

According to different characteristics of drivers, the paper selects some benchmarks to evaluate the performance loss regarding drivers' normal operations caused by the improved Chariot. Because Chariot is a mechanism inside the VM, all tests are done in* KVM* VM. The test is performed on a computer* Intel Core (TM)2 CPU 6300 at 1.86 G and 4 GB of RAM*; the network card type is* Realtek Semiconductor. Ltd. RTL-8139/8139C/8139C+*. Both the host and guest OS are* Centos 5.8*, and the* KVM* runs with* QEMU1.2*. Network test is accomplished by two identical computers.

The paper uses NATIVE to represent the performance of a driver's normal operations when a driver is not isolated and uses DWC (as outlined in Algorithm 1) and SCDWC (as outlined in Algorithm 2) to represent the performance of an isolated driver when Chariots, respectively, use different write operation capture algorithms.

#### 5.1.1. Network Drivers

Network drivers' performance overhead is measured by the* netperf* tool to test the sending and receiving performance of TCP and UDP. In the TCP test, the sending and receiving buffer is 16384 and 87380 bytes, respectively, and the message size is 16384 bytes. In the UDP test, the sending and receiving buffer is both 109568, and the message size is 65507.

As shown in Tables 1 and 2, when* e1000* network driver is isolated by Chariot with DWC algorithm, its throughput loss is quite significant, which is less than 1% the throughput of NATIVE, and the CPU utilization also reaches the saturation level. In this case, the driver performance is unacceptable, and the practicability of Chariot is also greatly reduced. However, using SCDWC algorithm can greatly improve the performance. From the throughput of different benchmarks, their performance is close to that of NATIVE, especially the TCP throughput. From CPU utilization of SCDWC algorithm, we can see that the driver's CPU utilization is close to that of NATIVE too. Meanwhile, when* rtl8139* network driver is isolated by Chariot with DWC algorithm, its throughput loss is also quite significant, and the CPU utilization also reaches the saturation level. After using SCDWC algorithm, the performance is also greatly improved and has a similar result with* e1000* driver.

#### 5.1.2. Disk Drivers

We run* tar* benchmark of uncompressing a file in a U disk and a scsi disk to measure the performance loss of the* usb_storage* and* sd_mod* driver. As shown in Tables 1 and 2, the performance loss of two different disk drivers which are isolated by Chariot with DWC algorithm is not as significant as network drivers but is still very large, they are less than 20% of NATIVE, and the CPU utilization is increased a lot. After using SCDWC algorithm, whether from the view of throughput or CPU utilization, drivers' performance is greatly improved, especially* usb* driver. Its throughput is near to that of NATIVE with an acceptable increase of CPU utilization.

#### 5.1.3. Sound Drivers

The sound driver benchmark uses the* mplayer* to play an MP3 file at a rate of 128 kbit per second. As shown in Tables 1 and 2, the performance loss of two different sound drivers which are isolated by Chariot with DWC algorithm is significantly less than 10% of NATIVE. Their CPU utilization also increases a lot and nearly reaches the saturation level. After using SCDWC algorithm, performance of both sound drivers is greatly improved, which have nearly achieved the performance of NATIVE. And their performance loss is very small and even can be ignored.

Since the optimal parameter of SCDWC algorithm is based on experiments of* e1000* network driver, performance of a few drivers still has some distance to that of NATIVE. However, this can be solved by similarly studying optimal parameters for those specific drivers. In conclusion, SCDWC algorithm has greatly improved the performance of isolated drivers; some are even close to that of NATIVE. As a result, the feasibility of Chariot is also greatly improved.

### 5.2. Reliability

**Figure 8:**
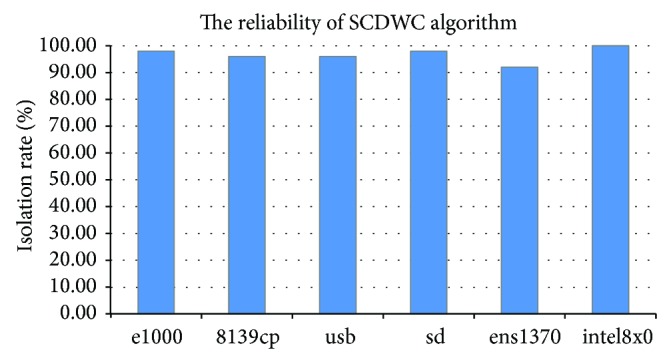
The reliability of Chariot after using SCDWC algorithm.

In order to fully measure the impact to the reliability of Chariot using improved algorithm, the paper tests 6 different isolated drivers with 50 injection faults. First, for 6 different drivers, we choose 50 injection faults which can be successfully isolated DWC algorithm. Then, we test the isolation efficiency in Chariot with SCDWC algorithm and record its isolation rate. The faults are injected by a common fault injection tool [9, 10]. The result of 300 fault injection tests of 6 drivers is shown in Figure 8. As shown in Figure 8, the isolation rate of injection faults with SCDWC algorithm has reached 90%. On the whole, the improved algorithm retains Chariot's good isolation property, achieving the design purpose. In another word, Chariot's reliability does not fall too much when improving its performance.

## 6. Related Work

Xen [6], L4Ka [5], and iKernel [7] use the virtualization technology to solve drivers' reliability problem. They isolate a driver and its kernel into an independent VM instance and use the isolation property between VM instances to isolate a driver. However, this method brings a complex problem of sharing devices. Different device drivers are often isolated in different VMs, especially bus drivers. This greatly increases the communication between VMs. In order to ensure the normal communication between different VMs and the host machine, especially the high-speed data transmission device, the memory sharing technology is often used to optimize performance. In addition, running an individual VM instance for each driver causes users to rent more resources to configure their running environment. Though some methods (such as page sharing, exchanging unused pages to the hard disk, and special treatment to zero page) can save some resources, they also bring the performance impact on users' virtualization environment. Relatively speaking, Chariot's optimization is through optimizing the capture of write operation to avoid too many traps to VMM. It does not change the original data processing of drivers in the VM and avoids increasing the complexity of driver isolation. Therefore, there is no need to use these technologies to optimize drivers' performance.

Palladium [11] uses the hardware segment protection mechanism to isolate kernel mode or user-mode extensions, and SIDE [12] extends it to isolate a network driver. However, using the segment protection mechanism makes the programming model of this method very complex and needs a lot of changes to the kernel. In order to avoid the excessive performance loss, a lot of optimizations are needed, and even the running process of the kernel is changed. For example, in order to avoid the TLB refresh, SIDE needs to intercept driver calls, and the page table of a driver is mapped to the user space of the kernel page table; in order to reduce the times of interactions between the kernel and drivers and avoid switching protection domains, a lot of kernel data are needed to be preallocated in drivers' local stack, which also brings data synchronization issues. Even in order to avoid triggering protection exception, the interruption is needed to be disabled, the instructions of system calls are needed to be replaced, and the privileged I/O instructions should be reduced. Though these optimization methods can obtain acceptable performance of the isolated driver, they are too complex and are not conducive to be extended to isolate new drivers. However, the performance optimization method of Chariot is aimed at the shadow page table in the VMM, does not need to modify the VM kernel, avoids increasing the complexity, and can be common to all drivers.

FPD [13] uses the hardware page protection mechanism to isolate user-mode extensions and provides the judgment strategy to determine whether various system calls in user-mode protection domain are legal or not. However, the method cannot isolate kernel mod extensions. In order to avoid TLB refresh brought by different page table switching, it uses the SPARC or Alpha hardware to mark TLB. At the same time, it uses the Window Invalid Mask (WIM) to reduce register refreshes, in order to avoid too large performance loss. Nooks [10] extends the page protection mechanism to the x86 architecture and creates private page tables for drivers to limit their write permissions through a complexity of synchronization and update mechanism. Because of using private page tables to isolate drivers, every interaction between the kernel and drivers needs to switch their page tables and stacks, which frequently refreshes the TLB and brings a great performance loss. Therefore, Nooks changes the memory management of drivers and tries to allocate needed data structures in the local stack if possible. In addition, interactions between the kernel and drivers are reduced by disabling interruptions and batching calls technologies and so forth. Then the performance loss of driver caused by the page table switching is reduced. However, these optimization methods need to largely modify the kernel, which makes its development too difficult and is not conducive to isolate new drivers. Relatively, Chariot does not depend on specific hardware architecture to obtain good performance. Although Chariot also uses the page protection mechanism, it moves the page protection down to the VMM; then its performance optimization just needs to be processed in the VMM. Therefore, we can avoid modifying the complex and changeful VM kernel and can easily achieve a good performance improvement.

Mondrix [9] uses a hybrid method of hardware and software isolation technologies, which provides 32-bit fine-grained permission control and accessing control for protection domains. Mondrix designs a large number of special caches in new hardware architecture. They are used to improve the matching rate of permission checks and avoid the TLB refresh problem when switching page tables. In addition, Mondrix reduces the memory overhead of permission tables for protection domains by refined designing these tables. It also uses the memory remap technology to eliminate the data copy between the kernel mode and the user mode, which maps discrete memory to a contiguous image and further improve the performance. However, these methods are complex and need a special hardware and to modify the kernel. Meanwhile, its isolation method and performance optimization methods lack commonality and are disadvantaged for its popularizing. Using Chariot, there is no hardware requirement, and it is simple and efficient. It can also be used in other page protection mechanisms for write operation capture. Nevertheless, Mondrix's permission table is a complement to Chariot's ACT to reduce the memory overhead.

XFI [14] uses inline guards and verifier to verify the correctness of behaviours and can isolate kernel extensions in a low performance overhead. It subtly designs different permission tables for different permissions and improves the performance by methods, such as avoiding data copying by wrappers. However, it cannot be used for complex kernel extension of traditional OS. BGI [15] extends XFI by manual intervening interfaces between the kernel and drivers, in order to isolate drivers with more complex interfaces. Its access control list can realize a variety of driver permissions' verification. Through reasonable building shared access control lists and conflict control lists, the access control list can be quickly queried. At the same time, using the shared access control lists can avoid wasting memory space. However, it depends on a good defined API of Windows driver model. LXFI [16] further extends BGI to a complex Linux driver interface and divides privileges of shared modules by proxies. In order to simplify the run-time checks, LXFI tracks proxies who obtain the written permission and checks whether a proxy has the write permission, in order to avoid some costly function pointer checks. Such software isolation methods primarily optimize run-time checks to improve performance, which is different to the optimization method of Chariot's write operation capture and may be referred to optimize Chariot's write operation check. Meanwhile, the design of their control lists can also be used to improve and optimize Chariot's ACT, thereby further reducing the query time and the space overhead of memory.

Many systems move drivers to a separate process address space, such as microkernels [17–19] and user-mode drivers [20–22]. In this way, driver faults just crash a corresponding user-mode process and do not cause the system crash. However, these methods are incompatible with traditional monolithic kernels, requiring rewriting all drivers, and cannot be widely applied. In addition, the microkernel technology has low efficiency and large amounts of data need to be frequently transferred between user-mode and kernel-mode, especially to high-speed device which causes seriously delay and performance loss. In recent years, there are a lot of optimization technologies to improve the performance of microkernel technology, including the IPC technology (such as asynchronous notification enhancement IPC and virtual message registers), the resource management technology (such as recursively page mapping and user-mod controlled kernel memory), and the schedule technology (such as the lazy schedule and directly processes switching). However, these technologies are always complex because their optimizations contrapose the difference of the traditional monolithic architecture. Relatively, Chariot does not need to do such optimizations. It just needs to simply optimize the capture of write operation, which can be much easier to get a close performance to original drivers while ensuring Chariot's reliability.

## 7. Conclusion

The driver fault greatly threatens the reliability of OS; thus much of reliability architecture is proposed to solve this problem. However, how to trade off the reliability and performance property is also a worth studying problem. This paper mainly studies how to improve the capture efficiency of drivers' write operation on the basis of ensuring the isolation property of Chariot and proposes an improved algorithm, SCDWC. Chariot sets shadow pages' write permissions to read-only to capture and check the write operations of isolated drivers and just opens a shadow page's write permission in a short time for a legal write operation. If a driver has large write operations, the write permissions of frequently accessed shadow pages are repeatedly changed which brings a great performance loss. With SCDWC algorithm, Chariot can cache the write permission of recently used shadow page, so as to decrease the isolated drivers' performance loss. The paper also studies the relationship between the number of shadow page caches and the performance of an isolated driver and infers the optimal number of shadow page caches which can well balance the reliability and performance property. The experimental result shows that SCDWC algorithm can greatly improve the performance of Chariot and also ensure its isolation efficiency under the optimal number of shadow page caches. As the future work, we will further improve the algorithm to achieve a better performance and study the difference trends between TCP and UDP experiments when changing the sizes of shadow page caches.

## Figures and Tables

**Algorithm 1:**
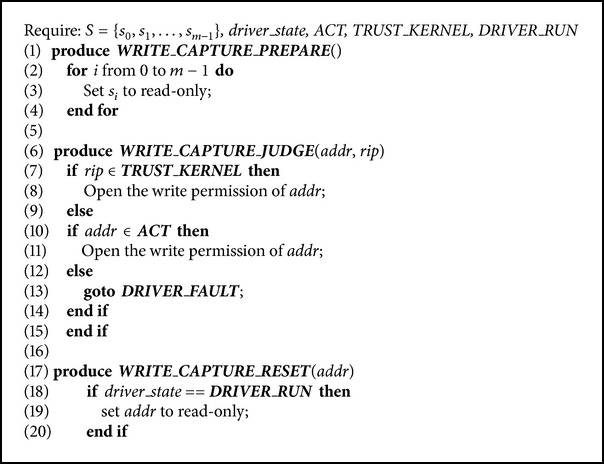
Traditional algorithm of the driver's write operation capture (DWC).

**Algorithm 2:**
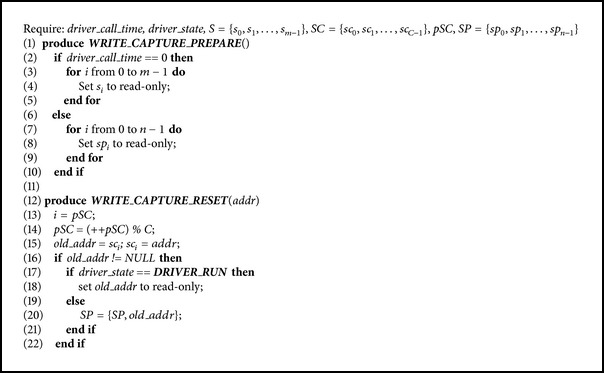
The algorithm of shadow page cache for the driver's write operation capture (SCDWC).

**Table 1:** The throughput (MB/s) or running time (s) of drivers, in the bracket is the relative performance; the performance of ens1370 and intel8x0 is running time; others are throughput.

Drivers	Benchmarks	NATIVE	DWC	SDWC
e1000	TCP-sending	93.95	0.47 (0.50%)	90.61 (96.45%)
	TCP-receiving	92.90	0.56 (0.60%)	92.28 (99.33%)
	UDP-sending	100.81	0.45 (0.45%)	60.85 (60.36%)
	UDP-receiving	92.47	0.77 (0.84%)	77.3 (83.59%)

rtl8139	TCP-sending	71.64	0.08 (0.11%)	39.69 (55.40%)
	TCP-receiving	94.05	0.21 (0.23%)	83.53 (88.82%)
	UDP-sending	122.54	0.11 (0.09%)	63.66 (51.95%)
	UDP-receiving	96.11	0.1 (0.10%)	36.1 (37.56%)

usb	tar	1.22	0.15 (12.69%)	0.99 (81.45%)

sd	tar	1.74	0.28 (16.11%)	1.10 (62.76%)

ens1370	mplayer	79.50	1094.2 (7.27%)	79.76 (99.68%)

intel8X0	mplayer	79.44	1318.3 (6.03%)	79.77 (99.59%)

**Table 2:** The CPU utilization (%) of drivers, in the bracket is delta CPU utilization according to NATIVE.

Drivers	Benchmarks	NATIVE	DWC	SDWC
e1000	TCP-sending	28.10	99.60 (+71.50)	46.62 (+18.52)
	TCP-receiving	49.45	99.79 (+50.34)	79.93 (+30.48)
	UDP-sending	50.29	99.71 (+49.42)	51.74 (+1.45)
	UDP-receiving	44.49	99.97 (+55.48)	56.66 (+12.17)

rtl8139	TCP-sending	50.23	99.95 (+49.72)	51.97 (+1.70)
	TCP-receiving	50.06	99.45 (+49.39)	52.69 (+2.63)
	UDP-sending	50.31	99.70 (+49.39)	51.18 (+0.87)
	UDP-receiving	37.86	94.06 (+56.20)	52.22 (+14.36)

usb	tar	3.75	35.18 (+31.43)	27.69 (+23.94)

sd	tar	16.22	39.08 (+22.86)	33.59 (+17.37)

ens1370	mplayer	0.0967	41.72 (+41.6233)	0.31 (+0.2133)

intel8X0	mplayer	0.0867	96.97 (+96.8833)	0.57 (+0.4833)
